# A comprehensive value-based method for new nuclear medical service pricing: with case study of radium [223^Ra^] bone metastases treatment

**DOI:** 10.1186/s12913-024-10777-8

**Published:** 2024-03-29

**Authors:** Haode Wang, Hui Sun, Yuyan Fu, Wendi Cheng, Chunlin Jin, Hongcheng Shi, Yashuang Luo, Xinjie Xu, Haiyin Wang

**Affiliations:** 1 Shanghai Health Development Research Center, (Shanghai Medical Information Center), Minhang District, No. 181 Xinbei Road, Shanghai, 201199 People’s Republic of China; 2 School of Health and Related Research (ScHARR), The University of Sheffield, Sheffield, S10 2TN United Kingdom; 3 National Health Commission Key Laboratory of Health Technology Assessment, School of Public Health, Fudan University, Shanghai, 200032 China; 4 Department of Nuclear Medicine, Zhongshan Hospital, Shanghai Medical College, Department of Nuclear Medicine, Shanghai Cancer Center, Fudan University, Shanghai, 200032 China; 5 School of Rehabilitation Medicine, Shandong University of Traditional Chinese Medicine, Jinan, 250355 China

**Keywords:** Service price, Health service, Value-based method, Health reformation, Fee-for-service

## Abstract

**Importance:**

Innovative nuclear medicine services offer substantial clinical value to patients. However, these advancements often come with high costs. Traditional payment strategies do not incentivize medical institutes to provide new services nor determine the fair price for payers. A shift towards a value-based pricing strategy is imperative to address these challenges. Such a strategy would reconcile the cost of innovation with incentives, foster transparent allocation of healthcare resources, and expedite the accessibility of essential medical services.

**Objective:**

This study aims to develop and present a comprehensive, value-based pricing model for new nuclear medicine services, illustrated explicitly through a case study of the radium [223Ra] treatment for bone metastases. In constructing the pricing model, we have considered three primary value determinants: the cost of the new service, associated service risk, and the difficulty of the service provision. Our research can help healthcare leaders design an evidence-based Fee-For-Service (FFS) payment reference pricing with nuclear medicine services and price adjustments.

**Design, setting and participants:**

This multi-center study was conducted from March 2021 to February 2022 (including consultation meetings) and employed both qualitative and quantitative methodologies. We organized focus group consultations with physicians from nuclear medicine departments in Beijing, Chongqing, Guangzhou, and Shanghai to standardize the treatment process for radium [223Ra] bone metastases. We used a specially designed ‘Radium Nuclide [223Ra] Bone Metastasis Data Collection Form’ to gather nationwide resource consumption data to extract information from local databases. Four interviews with groups of experts were conducted to determine the add-up ratio, based on service risk and difficulty. The study organized consultation meeting with key stakeholders, including policymakers, service providers, clinical researchers, and health economists, to finalize the pricing equation and the pricing result of radium [223Ra] bone metastases service.

**Main outcomes and measures:**

We developed and detailed a pricing equation tailored for innovative services in the nuclear medicine department, illustrating its application through a step-by-step guide. A standardized service process was established to ensure consistency and accuracy. Adhering to best practice guidelines for health cost data analysis, we emphasized the importance of cross-validation of data, where validated data demonstrated less variation. However, it required a more advanced health information system to manage and analyze the data inputs effectively.

**Results:**

The standardized service of radium [223Ra] bone metastases includes: pre-injection assessment, treatment plan, administration, post-administration monitoring, waste disposal and monitoring. The average duration for each stage is 104 min, 39 min, 25 min, 72 min and 56 min. A standardized monetary value for medical consumables is 54.94 yuan ($7.6), and the standardised monetary value (medical consumables cost plus human input) is 763.68 yuan ($109.9). Applying an agreed value add-up ratio of 1.065, the standardized value is 810.19 yuan ($116.9). Feedback from a consultation meeting with policymakers and health economics researchers indicates a consensus that the pricing equation developed was reasonable and well-grounded.

**Conclusion:**

This research is the first study in the field of nuclear medicine department pricing methodology. We introduce a comprehensive value-based nuclear medical service pricing method and use radium[223Ra] bone metastases treatment pricing in China as a case study. This study establishes a novel pricing framework and provides practical instructions on its implementation in a real-world healthcare setting.

## Introduction

Pricing medical services is a critical task in the era of rapid advancements in medical technology and service pattern for all providers [1–4]. This is especially true for the nuclear medicine department, which offers some of the most costly and complex services. Although many public healthcare system or dominated payers reimburse the service providers by cost containment methods, such as negotiated contractual fee rate [5], per capitation arrangements, Diagnostic Related Group [6], Diagnosis-Intervention Packet (DIP) [7] and bundle payment, the FFS model remains the most viable option for reimbursing innovative services in the early stages, including those provided by nuclear medicine departments. FFS provides straightforward incentives for promoting the new technology application, rewarding medical professional training [6] and laying the groundwork for future bundle payment [8]. For instance, following the implementation of the ‘zero markup’ drug price policy across all Chinese tertiary hospitals in 2017, service fees have become the only option to compensate the time spent on providing the service [9].

In most reimbursement systems, the FFS costs are calculated in a cost-based way rather than value-based [8]. Time-driven activity-based costing (TDABC) is often considered as ‘gold standard’ in cost-based method. This approach calculates treatment path costs by identifying the cost of each activity [10], with indirect costs averaged over time units. Conversely, the direct cost calculation method focuses solely on the direct costs (excluding the sharing cost of management and operations), excluding indirect costs entirely [11]. While TDABC is a bottom-up process providing reliable result if hospital accounting system accurately classified and recorded essential indirect costs [12], it may not fully accommodate the requirement of innovative services pricing due to facility sharing, inappropriate administration fee estimation and underestimated management time such as license processing. Direct costing method, despite the fact that it overlooked the indirect cost, identifies significant cost factors and offers transparent cost calculation framework with a project-specific cost dashboard under changing treatment environment [13, 14], making it potentially more practical for new treatment pricing.

For nuclear medicine department, one issue with cost-based healthcare service pricing is that it overlooks value factors inherent in service procedures. For instance, health professionals and patients may be exposed to extra risks, such as shots and radiation [15] during delivering the innovative service, and handling innovative radiopharmaceuticals requests certification and extra training [16], which needs more time investment. These aspects should be thoroughly considered in pricing. Value-Based Health Care (VBHC) has gained global attention [17]. One example is the China National Healthcare Commission advocates for pricing healthcare service with value add-up ratio [18]. Nevertheless, VBHC methods have faced challenges, including transparency concerns and communication issues between policymakers and clinical professionals [3].

To address challenges mentioned and serve the pricing needs in the nuclear medicine department, this study introduces a comprehensive value-based pricing method. Recognizing that innovative treatments vary across different environments and countries, our approach avoids focusing on uncertain or unpredictable medical environment [4]. We stick with the value-based pricing framework, developed by health economics researches, suggesting compensating effective new therapies with ex ante risk prediction at the innovation promotion stage [19–21]. The method considers direct costs, technical difficulty, and technical risk (both patient and clinician) of new services for calculate a standardized price. Apart from the cost of providing this service, we constructed a relative price ratio by comparing the difficulty and risk of new treatment with existing services, hypothesizing that an add-up ratio can reflect the innovation’s value [18]. This methodology, exemplified through the radium [^223^Ra] bone metastases treatment pricing study, is applicable for public-funded healthcare system but also feasible for private payers to calculate a reasonable price.

## Methods

### Value-based pricing equation

The *National Fee Schedule* published by China National Healthcare Commission advocated a cost-based method for healthcare service pricing since 2001 [22, 23]. In 2012, the new version of *National Fee Schedule* recommended to consider Resource-Based Relative Value Scale (RBRVS) for existing medical service item pricing [23, 24]. This update introduced a medical risk number and a technical difficulty number for each service listed. However, the document neither introduced the guideline on using the factors nor the strategy for attaching scores to new service. Haiyin et al. [18] developed a value parity model that calculated service standardized price with risk and difficulty numbers for traditional Chinese medicine service price in Shanghai.

Building on this conceptual framework, our research has refined and expanded the standardized price calculation equation to new service pricing in the nuclear medicine department. The equation calculates a service standardized value (Eq. 1), which comprises two components: the standardized professional service value (Eq. 2) and the standardized resource consumption value (Eq. 3). The standardized professional service value represents the manpower invested in the service process multiplied by the service add-up ratio. The resource consumption value calculates the direct resources is determined using the Activity-Based Costing method [25], focusing on the direct resources consumed and excluding the administration expenses, logistics, financial costing, and real estate depreciation.

There are three value factors requested in the two equations: the human resource input (including physicians, nurses, nuclear medicine technicians and other supporting professional), medical consumables and direct material cost, and service add-up ratio (reflecting relative technical difficulty and risk score). By aggregating the standardized professional service value and the resource consumption value, we derive the standardized value of an innovative nuclear medicine department service. The standardized value forms the basis of our suggested service price for discussion.

Equation 1: standardized value calculation equation.

$$\begin{array}{l} P={Y}_{Standarized\,professional\,service\,value}\\+{C}_{Standarized\,\text{r}\text{e}\text{s}\text{o}\text{u}\text{r}\text{c}\text{e}\,\text{c}\text{o}\text{n}\text{s}\text{u}\text{m}\text{p}\text{t}\text{i}\text{o}\text{n}\,\text{v}\text{a}\text{l}\text{u}\text{e} }\end{array}$$

#### Note

*P is the standardized value for any service; Y is the standardized service value; C is the standardized resource input. The Y and C shares the same meaning in Eqs. 2 and 3.*

Equation 2: Standardized professional service value.

$$\begin{array}{l} Y=\left(\sum _{i}^{n}{M}_{i}\times \frac{{X}_{i}}{Annual\,working\,minutes}\times {T}_{i}\right)\\\times\,\left(1+Lg\sqrt{\frac{New\,service\,difficulty\,score \times New\,service\,risk\,score}{Baseline\,service\,difficult\,score\times Baseline\,service\,risk\,score}}\right)\end{array}$$

#### Note

*Assume that there are n kinds of health professional participated in a treatment. Xi is the related health professional i annual income level; Mi is the number of health professional i participated in; Ti is the time each health professional i spent on the new service every time;* $1+Lg\sqrt{\frac{New\,service\,difficulty\,score \times New\,service\,risk\,score}{Baseline\,service\,difficult\,score\times\,Baseline\,service\,risk\,score}}$ *is the service add-up ratio. This should not include any administrative members.*

Equation 3: Standardized resource consumption value.

$$ C={\sum }_{i}^{m}{K}_{i}\times {P}_{i}$$

#### Note

*Assume that there are m kinds of medical consumables (not including drugs or anything that charged separately) used in the service. Ki is the number/unit/time of medical consumable i used; Pi is the unit cost of medical consumable i. Administrative cost should not include but the preparing cost should be counted.*

### Research design and data source

The services pricing had six main steps. The initial step was to define the standardized procedure for the administration and follow-up of service (the radium [^223^Ra] bone metastases treatment). This definition was established through an extensive literature review and key expert consultations. Radium [^223^Ra] bone metastases treatment was an intravenous injection service with the Radium-223 dichloride (radium [^223^Ra]) medicine [26, 27] for patients with metastatic castrate-resistant prostate cancer (mCRPC) and symptomatic bone metastases [26–29]. A review of treatment guidelines in other countries [28, 29] and comparison with medicine administration guideline in China was conducted. This step was crucial to avoid any potential double-counting of time. Following this, we initiated a series of consultations with senior doctors of nuclear medicine departments, which had been approved for radium [223Ra] bone metastases treatment since December 2020. The primary objective of these consultations was to verify that the service stages defined were clear and in alignment with actual clinical practices.

The second step was to develop data collection instruments. These instruments were aligned with the cost calculation guideline and guided by insights drawn from the ‘*New Medical Service Cost Schedule’* and the cost components in ‘*2012 Manual on the Prices of Medical Procedures.’* We developed ‘*Radium [*^*223*^*Ra] Bone Metastases Treatment New Medical Service Cost Collection Forms’*. The instrument consisted of five distinct forms where each focused one specific type of data input: human resource input, salary levels, medical consumables used, unit price of medical consumables, and the current pricing of Radium [^223^Ra] Bone Metastases Treatment service. As mentioned, our approach adhered to the principles of activity-based costing and only direct costs considered.

The third step was data collection through interview, focus group and clinical time recordings. The input data collection should be conducted in all centers or key centers to maintain a good representativeness. To gather both retrospective and prospective data, we employed a hybrid method, combining consultations and clinical time recordings. This hybrid method allowed cross validation and mitigated possible recall bias [30]. We organized three focus group and clinical time recordings in Beijing, Chongqing and Guangzhou in 2021. During the focus groups, we engaged with the department healthcare professionals who were requested to compete sections of data collection forms. A following clinical time recording was conducted by two trained researchers to record the time spent by each health professional on each procedure through their health database. This hands-on time recording was integral in providing us with a real-time perspective of the treatment process, complementing the data obtained through other methods. Further unit price data was extracted from the Hospital Information System (HIS) to convert the unit cost into monetary terms.

The fourth step was to find the add-up ratio by determining the difficulty, risk score and the baseline treatment. However, all of the new service encountered the challenge that there was neither risk and difficulty scores in the published service fee schedule, nor designated baseline treatment for nuclear medicine department. To address this gap, we employed Delphi method [31] to consult with experts and health economists. During the consultation interview, we provided a comprehensive overview of the published 2012 National Fee Schedule, particularly focusing on its criteria for setting risk and difficulty scores. All of the services in same category (bone metastases and nuclear treatment) were listed. Participants were asked to evaluate services based on several criteria: proficiency required, similarity to the targeted treatment, satisfaction with current price, and the proportion of human resource input. Then three baseline treatments were suggested by each participant. Their task was to identify the top-three similar treatment, treatment with slightly lower difficulty/risk and treatment with slightly higher difficulty/risk compared to the radium [^223^Ra] treatment. After thorough deliberation, the research team selected the most appropriate existing service as the baseline and decide risk/difficulty score for new service.

**Fig. 1:**
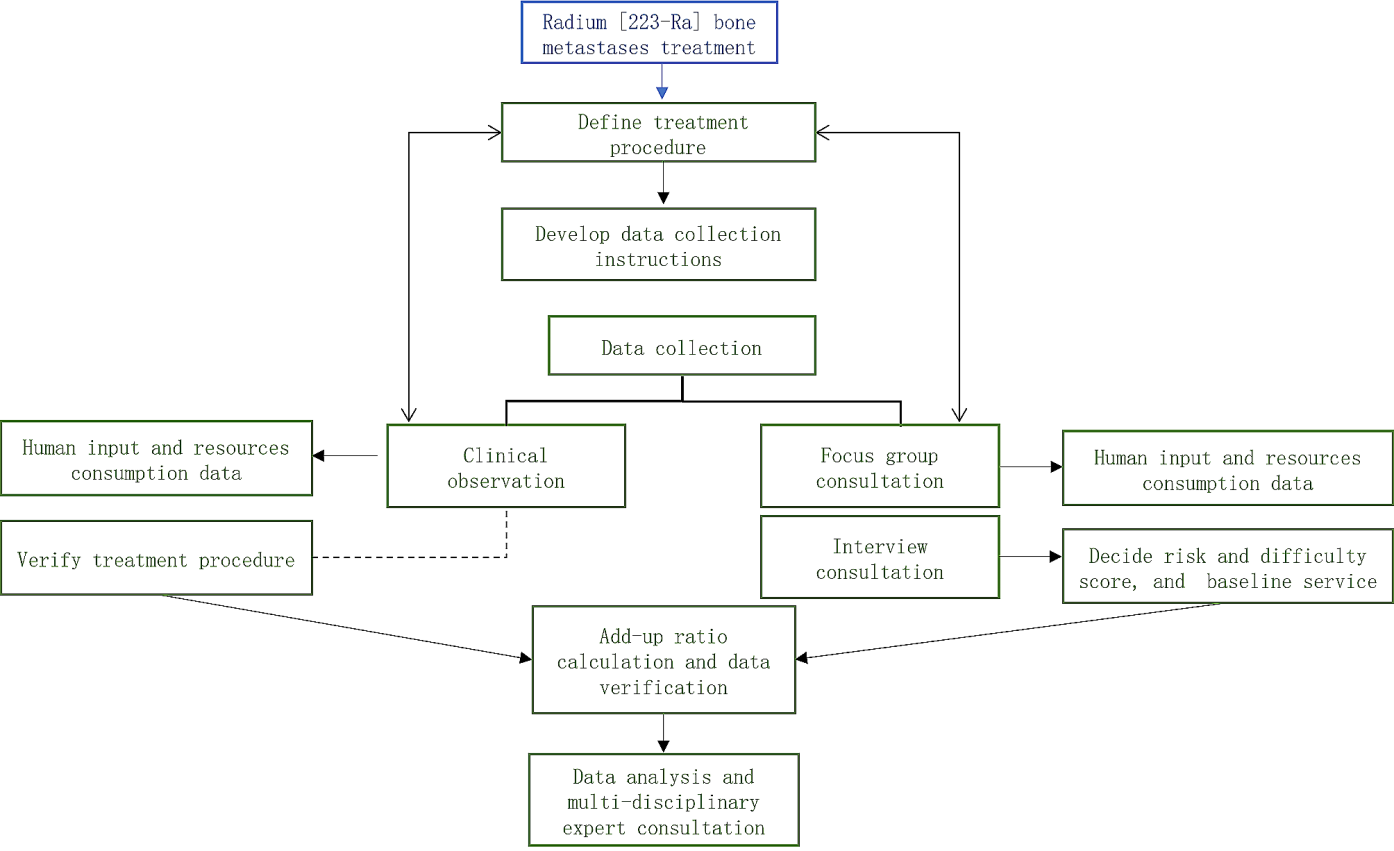
Radium [^223^Ra] bone metastases treatment pricing research flow chart

The fifth and sixth steps were data analysis and result presentation on multi-disciplinary consultation meeting. Data from various centers were summarized to derive the average value or median value, depending on the standard deviation, for calculating standardized value. The last stage of our project involved a comprehensive consultation meeting where we presented the gathered cost data, outcomes from the qualitative consultations, the national reference standardized price, and the details of our pricing instrument to interdisciplinary stakeholders. This meeting convened experts in the field of nuclear medicine departments, health economics, health policy, industry and pharmaceutical companies, and hospital finance. It was necessary to validate the findings and methodologies before publish the result (Fig. 1).

### Data analysis and outcome consultation

All the cost data was digitalized and analyzed with Microsoft^@^ EXCEL 2019 and StataMP 17. The qualitative data was analyzed through thematic analysis method with Microsoft^@^ WORD 2019 software [32]. In order to present the pricing equation in a digitalized format, we used Microsoft^@^ EXCEL 2019 to develop universal pricing instrument to assist local price calculation and standard deviation calculation.

## Results

### Standardized treatment process

The 2012 *National Fee Schedule* defined the bone metastases treatment with radio isotope as *the overall treatment service from clinical history looking up to nuclear waste management*. To avoid overlap in the treatment procedure, we excluded time spent on cancer diagnosis in other departments and time spent on the patients who did not proper for the treatment. Consequently, the treatment service was defined as *medical activities related with qualified patients including disease evaluation, drug injection and nuclear waste management (if applicable). The cost will not consider any extra cost related to unqualified patients, drug waste, follow-up, cancer diagnostic fee paid separately and administration cost for drug license (due to China customs regulation).*

In line with clinical practices and existing literature, we divided the treatment into five independent procedures: pre-treatment evaluation (involving a multi-disciplinary team meeting to strategize the isotope injection and review the efficacy of previous treatments), treatment planning (nuclear radiologist or other qualified professionals assessing the dosage and calculates the amount for injection), drug injection (preparing the injection environment and administering the drug dose via slow intravenous injection), post-injection on-site observation (removing the syringes and monitoring for potential side effects) and nuclear waste management (storaging, disposing and handling for radioactive residuals).

### Input data collection

Clinical input data was gathered from three leading medical institutes in Beijing, Chongqing and Guangzhou (Table 1). We evaluated the reliability of the data collected on human resource input and the cost of medical consumables from multiple sources of data. In Beijing, the total human resource input for a single treatment was 252 min and the most time-intensive procedure was post-injection on-site observation. In contrast, the medical professionals in Chongqing’s nuclear department devoted a considerably longer duration, totaling 402 min per treatment. The pre-treatment evaluation was the most time-consuming. Institute in Guangzhou spent 239 min on average while the pre-treatment evaluation procedure was the longest as well. When translating these time inputs into monetary terms with local average wages of medical professionals, the human input in Beijing, Chongqing and Guangzhou (Table 2) were ¥703.44 ($100.5), ¥711.61 ($101.1) and ¥731.38 ($104.5). Our research identified ten types of disposable medical consumables used in the treatment, with an average cost of ¥54.94 ($7.85) per session.

**Table 1:** Radium [^223^Ra] bone metastases treatment pricing research consultation participants

	Nuclear department technologist and physician^1^			Clinical nurse			Other participants^2^	Total number
	Deputy Chief and Chief	Physician/technologist	Healers	Nurse Director / Deputy Director	Nurse in Charge	Nurse		
Define treatment procedure	3	0	0	0	0	0	1	4
Data collection	4	1	1	1	2	2	2	13
Add-up ratio calculation	5	0	0	0	0	0	1	6
Multi-disciplinary expert consultation	12	0	0	0	0	0	7	19

*Note: 1. due to the position name difference in different hospitals, nuclear department technologist includes physical therapist, chemotherapist, radiologist and other positions if applicable*

*2. Other participants includes health economists, policymakers, pharmaceutical company representatives and hospital financial experts*

**Table 2:** Radium [^223^Ra] bone metastases treatment input, variation and coefficient of variation

		Total human input (min)	Pre-treatment evaluation		Treatment planning		Drug injection		Observation		Waste management		Total human input ($)		Pre-treatment evaluation	Treatment planning	Drug injection	Observation	Waste management	Medical consumable cost ($)	
Beijing		251.50	52.00		38.00		27.50		84.00		50.00		100.49		27.06	12.87	4.53	41.94	14.09	6.63	
Chongqing		402.00	170.00		90.00		30.00		62.00		90.00		101.66		51.94	10.87	6.33	16.94	15.59	6.89	
Guangzhou		239.00	90.00		29.00		20.00		70.00		30.00		104.48		45.09	12.72	6.09	32.47	8.12	7.85	
Average value		297.50	104.00		52.33		25.83		72.00		56.67		102.21		41.36	12.15	5.65	30.45	12.60	7.37	
Standard Deviation		11.73			49.18		26.89		4.25		9.09		24.94		1.68	10.49	0.91	0.80	10.31	3.23	0.52
Coefficient of variation		0.25			0.47		0.51		0.16		0.13		0.44		NA	0.04	0.01	0.02	0.05	0.04	0.01

For variation, we observed that the human input in terms of time was more variable than when measured in monetary units and compared to the variability in consumables used. Pre-treatment evaluation, treatment planning and nuclear waste management had the largest variation among all the procedures. The S.D. of overall human input time was 11.73 and the coefficient of variation is 0.25, where treatment planning had the largest variation. Medical consumable cost had a S.D. of 0.52 and a coefficient of variation of 0.01 (Table 2). The qualitative interviews conducted as part of our study shed light on some potential reasons for these variances. One notable factor was the differences in treatment procedure arrangements that some institutes were likely to have more professionals to monitor the radiation dose. Additionally, the impact of the learning curve on nuclear medicine professionals’ treatment times was also identified as a significant factor influencing the variability [33].

Through the collective insights gathered from the focus groups and interviews, we arrived at a consensus regarding the scores for treatment difficulty and risk associated with the new treatment. It was determined that the new treatment difficulty score was 90 (out of 100) and the risk score was 95 (out of 100). Participated experts agreed that the new treatment had considerable complexity and hazards due to the new β nuclide used. The baseline treatment was identified as strontium [^89^Sr] bone metastases treatment. This baseline treatment has a slightly lower difficulty score of 80 and a risk score of 92. The service add-up ratio for the new service was calculated to be 1.065.

### Standardized value calculation

A mean human input value from data collected across three sample points, representing the standardized human input factor for radium [^223^Ra] bone metastases treatment (Table 3). The overall treatment time was calculated to be 298 min, distributed as follows: 104 (34.96%) minutes for pre-treatment evaluation, 52 (17.45%) minutes for treatment planning, 26 (8.41%) minutes for drug injection, 72 (24.20%) minutes for post-injection on-site observation, 56 (18.82%) minutes for nuclear waste management. The average human input in monetary term was ¥715.48 ($102.07), ranging from ¥703.44 ($100.5) to ¥731.38 ($104.48). We determined the standardized medical consumable cost by taking the median medical consumable cost, which was ¥48.20 ($6.91) [34, 35]. Thus, the standardized cost for radium [^223^Ra] bone metastases treatment, encompassing both the standardized professional service value and the standardized resource consumption value, was ¥763.68 ($109.10), with variations ranging from ¥757.76 ($108.25) to ¥786.32 ($112.33).

**Table 3:** The standardized professional service value and standardized resource consumption value of radium [^223^Ra] bone metastases treatment service

Procedure	Human input(min)	Rank	Standardized professional service value($)	Cost proportion percentage(%)	Total cost with standardized resource consumption value ($)
Pre-treatment evaluation	104.50	1	41.36	40.47	
Treatment planning	39.50	4	12.15	11.89	
Drug injection	25.50	5	5.65	5.53	
Observation	72.00	2	30.45	29.79	
Waste management	56.00	3	12.60	12.32	
**In total**	297.50		102.21	100.00	109.10

We multiplied the mean human input value by the service add-up ratio to calculate the standardized professional service value, mean human input value needs to time service add-up ratio. Based on recommendations from health economists and policymakers, the add-up ratio should not exceed 1.5 to maintain income balance. The add-up ratio for radium [^223^Ra] bone metastases treatment (1.065) was below 1.5 resulting in a standardized professional service value of ¥761.98 ($108.70), ranging from ¥749.16 ($107.45) to ¥778.92 ($111.27).

Consequently, the standardized value, obtained by adding the standardized professional service value to the standardized medical consumable cost, was ¥810.19 ($115.71), ranging from ¥804.10 ($114.91) to ¥833.86 ($119.12). The consultation meeting in early 2022 with multi-disciplinary experts suggested to use standardized value as the new treatment service suggested price.

## Discussion

Our study focuses on Fee-For-Service (FFS) pricing for innovative treatments, a common approach globally, yet with significant variability in reimbursement standards for nuclear medicine department services [36–38]. The diversity in international pricing practices underscores the impracticality of a universal price for a single service, where a transparent function with local input data should be more feasible [39]. In the nuclear medicine department, each service is distinct due to the procedure, radioactive nuclide used, and the relative risk for patients. A transparent pricing framework is necessary.

The pricing of healthcare services affects both provider behavior and patient decision-making. The World Health Assembly (WHA) recognizes its impact on the safety, affordability, and accessibility of universal health coverage [40]. Patient’s behavior and decisions are strongly influenced by latent price or real price of new service. Unreasonably high prices can deter patients, even those with severe conditions [41]. On the other hand, physicians may be afraid of losing participation right or autonomy in the process of value-based service pricing [42]. For both providers and service purchasers, inappropriate pricing can lead to market inefficiencies and health losses, with inadequate compensation possibly resulting in additional charges and over-compensation restricting equitable access to new technologies [6]. On the other hand, there is limited number of innovative healthcare service pricing research published and the majority of FFS pricing frameworks fail to consider the importance of service difficulty and risks, which are of great importance for nuclear department service providers.

China has been a forerunner in pricing new healthcare services within public-funded hospitals, moving towards a ‘zero-markup’ drug price policy in the future years [43]. Service prices becomes the primary source to compensate healthcare professional’s contribution. To establish a better service price system, the 2012 *National Fee Schedule* encourages considering additional value factors such as difficulty and risk. A joint report drafted by World Bank (WB) and World Health Organization (WHO) suggests HTA as an efficient way in supporting service quality control and provided a framework to consider the concept of value [44, 45]. This pricing research employs a HTA value framework by considering unambiguous value of new service (difficulty and risk) in new nuclear medicine department treatment pricing equation. To our knowledge, this study is the first study reported a systematic new treatment pricing equation for nuclear department service pricing. To prevent any data collection bias and practical issues, we organized a multi-disciplinary consultation to include opinions from medical professionals, healthcare service purchasers, healthcare institute accounting manager and third-party health economists. We took radium [^223^Ra] bone metastases treatment pricing research as an example to illustrate how to use the equation.

Our result revealed that the standardized cost for radium [^223^Ra] bone metastases treatment was $109.10 (with a range from $108.25 to $112.33), and the standardized value with add-up ratio was $115.71 (with a range from $114.91 to $119.12). Feedback from stakeholders and treatment time recordings highlighted significant procedural variations and a learning curve effect for this new service. For example, more experienced nuclear professionals tended to do patient talk after the nuclide injection and less people participate in the process. These variations did not undermine the critical need for clearly defined standardized procedures. Inaccurate procedure specifications decreased the reliability of cost data collection, weakened stakeholder engagement, and led to cost unit double-counting. We also demonstrated the feasibility of using a singular add-up ratio to reflect the relative value of new service. This approach aligned with the classic health economics models and straightforward for understanding [46, 47]. However, as stated above, the add-up ratio should be equitable, adaptable and effective for all. The qualitative process was important and must adhere to a transparent pre-determined guideline or steps.

It is worth notification that we have no intention to draw the opinionated conclusion that this pricing equation is the single best one for all healthcare system and for all departments. The primary goal of this pricing research is to introduce a systematic way of transparent value-based pricing and gather empirical evidence supporting this concept in the nuclear medicine department. Health technology assessment institutes and healthcare payers support value-based resources allocation in the field of new service payment. A notable example of its application is the assessment of orthopedics treatment with spinal surgery robot in UK, US and Thailand [48–50]. In both public healthcare and private healthcare sectors, curative and rehabilitative care accounts for over 60% of total healthcare expenditure [51, 52]. It is expected that using value-based pricing strategies for new service pricing is an effective strategy in curbing the rise in healthcare spending. This pricing equation can be tailored for any definition of ‘value’. Furthermore, it can be utilized for price adjustment by selecting serve prices deviated from the standardized values [18, 53].

Two primary challenges associated with the value-based pricing are defining the ‘value’ and collecting the evidence of value to support the pricing. Defining ‘value’ requests reaching a consensus among stakeholders, a process that demands considerable time. The latter, collecting evidence, can be resource-intensive and request a well-designed information system to support. For the nuclear medicine department in China, the interviewed healthcare professionals agreed that new service difficulty (related with minimum full-time training time) and risks (radiation dose) were the most inhibiting factors, a viewpoint also shared by Chinese policymakers. However, the concept of ‘value’ can differ across departments, legislations and countries. Researchers should prepare for an inconsistent opinion between payer and service provider on ‘value’. Collecting unbiased cost data mandates significant manpower and investment in survey and information system. Our research has shown that any data collection should be cross-validated to mitigate recall bias, which is prevalent when relying on a single data source. Future research should extend this equation to other services in multiple departments, and compare different ways of data collection if data is available.

This study has three limitations: first, although the research group collected data from all of the institutes administering radium [^223^Ra] bone metastases treatments in 2021, there was only four cities included. It is possible that secondary hospitals or tertiary hospitals in other cities may differ from samples in operation. Potential bias due to small sample might lead to an overestimation of the add-up ratio. Second, whether the price ensures a proper and acceptable compensation for service providers requires retrospective test, which is not possible at this stage. Without this retrospective evaluation, the fairness and practicality of the pricing model remain uncertain. Third, our approach to defining ‘value’ was predominantly from a policy-making and clinical standpoint, neglecting the patient’s perspective. Although elucidating the nuances of innovative treatments to patients can be complex, excluding patient satisfaction from the value framework might lead to criticism from patient organizations and health insurers.

## Conclusion

This research introduced a value-based pricing framework for innovative nuclear medicine department services, using the pricing of [^223^Ra] bone metastases treatment as a case study. The pricing function takes into account various factors, including the inputs required for treatment, the difficulty and risk associated with the procedure, and a baseline for standard treatment. A key feature of this pricing method is the inclusion of an add-up ratio, which is designed to quantify the relative human input value of the new service with one factor. The validity and effectiveness of this pricing framework were confirmed through a multi-disciplinary meeting. Although this study serves for nuclear medicine department services pricing, the framework offers a structured approach to value-based pricing across various medical services.

## Data Availability

Due to the large amount of data collected and large number of qualitative records, please contact Haiyin Wang (why0522@126.com) for accessing the full data.

## Declarations

### Additional information

Deidentified data will be made available to researchers in a HIPPA-compliant manner upon request for the purposes of replication.
